# Prospective evaluation of artificial intelligence (AI) applications for use in cancer pathways following diagnosis: a systematic review

**DOI:** 10.1136/bmjonc-2023-000255

**Published:** 2024-05-10

**Authors:** Sheba Macheka, Peng Yun Ng, Ophira Ginsburg, Andrew Hope, Richard Sullivan, Ajay Aggarwal

**Affiliations:** 1 Institute of Cancer Policy, King's College London Faculty of Life Sciences & Medicine, London, UK; 2 National Cancer Institute Center for Global Health, Bethesda, Maryland, USA; 3 Radiation Oncology, Princess Margaret Hospital Cancer Centre, Toronto, Ontario, Canada; 4 Dept of Health Services Research & Policy, London School of Hygiene and Tropical Medicine, London, UK

**Keywords:** radiation oncology, chemotherapy, palliative care, surgical oncology

## Abstract

**Objectives:**

To assess the clinical readiness and deployability of artificial intelligence (AI) through evaluation of prospective studies of AI in cancer care following diagnosis.

**Design:**

We undertook a systematic review to determine the types of AI involved and their respective outcomes with a PubMed and Web of Science search between 1 January 2013 and 1 May 2023.15 articles detailing prospective evaluation of AI in postdiagnostic cancer pathway were identified.

**Setting:**

The role of AI in cancer care has evolved in the face of ageing population, workforce shortages and technological advancement. Despite recent uptake in AI research and adoption, the extent to which it improves quality, efficiency and equity of care beyond cancer diagnostics is uncertain to date.

**Interventions:**

We appraised all studies using Risk of Bias Assessment of Randomised Controlled Trials (ROB­2) and Risk of Bias In Non­randomised Studies- of Interventions (ROBIN­I) quality assessment tools, as well as implementational analysis concerning time, cost and resource, to ascertain the quality of clinical evidence and real-world feasibility of AI.

**Results:**

The results revealed that the majority of AI oncological research remained experimental without prospective clinical validation or deployment. Most studies failed to establish clinical validity and to translate measured AI efficacy into beneficial clinical outcomes. AI research is limited by lack of research standardisation and health system interoperability. Furthermore, implementational analysis and equity considerations of AI were largely missing.

**Conclusion:**

To overcome the triad of low-level clinical evidence, efficacy-outcome gap and incompatible research ecosystem for AI, future work should focus on multi-collaborative AI implementation research designed and conducted in accordance with up-to-date research standards and local health systems.

**WHAT IS ALREADY KNOWN ON THIS TOPIC**

- The adoption of artificial intelligence (AI) in cancer care has gained traction in recent years, specifically in cancer diagnostics due to the emergence of high-quality real world evidence. However in general, most AI research studies remained preliminary and retrospective in nature, thus limiting its clinical deployability. Prior to this review, there was no systematic review focusing on studies prospectively evaluating AI beyond cancer diagnostics. Therefore, this review provides an up-to-date research landscape of AI in the postdiagnostic cancer pathway to determine its clinical readiness and deployability.

**WHAT THIS STUDY ADDS**

- From this review, we attested to the fact that only a small proportion of AI studies were prospective. Most of the prospective studies lack internal and external validities as they were single-site studies with small sample sizes. In addition to the low level of clinical evidence, the incompatible research ecosystem and the lack of implementation science research further hinders clinical deployment of AI.

**HOW THIS STUDY MIGHT AFFECT RESEARCH, PRACTICE OR POLICY**

- This review dissected and analysed the multifactorial implementation barriers of AI in real-world clinical setting. We formulated a point-by-point action plan targeted at different key stakeholders, specifically commissioners, academics and clinicians, to recommend them of future steps required to optimise clinical readiness and deployability of AI in cancer care.

##  Introduction

Cancer care is becoming more complex with demographically ageing populations, rising socioeconomic inequalities and the rapid development of novel technologies for treatment.^1 2^ This complexity coupled with healthcare workforce shortages and infrastructure deficits have created significant opportunities for artificial intelligence (AI) technologies to reshape cancer care across a range of domains and the patient pathway.^3^

To date, the impact of AI technology is most prominent in cancer diagnostics, particularly radiology.^4^ AI-based breast screening system has achieved non-inferior performance in interpreting mammograms when compared with expert clinicians in a real-world environment.^5^ Paige Prostate, an AI software that improves the accuracy and efficiency of prostate biopsy diagnosis, has also received Food and Drug Administration approval in the USA.^6^ In the field of cancer treatment planning, AI technology has also made significant inroads. OSAIRIS, an open-source AI in medical image analysis, was piloted in a UK hospital and proven to significantly shorten the time required for radiotherapy planning.^7^ Similar progress has been observed in other areas, such as patient monitoring, precision oncology, behavioural modification and treatment response prediction. For example, machine learning AI models have been incorporated into the evaluation of cell-free DNA advancing the development of liquid biopsy by increasing detection rate and improving monitoring of cancer.^8^

Nonetheless, progress in applying AI in oncology and healthcare has been tempered by uncertainties regarding the feasibility of AI integration into routine clinical pathways and the extent to which it actually improves the quality, efficiency and equity of cancer care.^9^ This is, in part, based on concerns regarding the lack of large-scale prospective evaluation of AI algorithms in diverse clinical settings to establish clinical robustness, resource savings and budget impact. AI algorithms are prone to biases that can negatively impact their performance, particularly when they are trained on inadequate, heterogeneous and retrospective datasets.^10^,^12^ The case of IBM Watson Health’s cancer AI algorithm is a relevant example, as many of the treatment options initially recommended for patients with cancer have been found to contain significant errors.^13^ Although it is currently clinically deployed in multiple countires, namely Brazil, China, India, South Korea and Mexico, the most recent concordance study in 2019 reveal varying level of disconcordance between AI-derived recommendations and standard.^14^

Furthermore, AI faces implementation barriers across very different health ecosystems around data security, antiquated or dynamic regulatory guidelines, administrative burden and lack of research standardisation.^15 16^ The potential for inequalities in healthcare delivery through the deployment of AI interventions has also been raised by WHO, as the design, development and delivery of AI runs the risk of widening existing disparities or entrenching biases.^17^

To the best of our knowledge, no systematic attempt has been made to assess the readiness and deployability of AI in oncology, beyond cancer diagnostics. Our systematic review focused specifically on evaluating the level of evidence for new AI solutions, specifically identifying studies that have sought prospective evaluation of AI tools in the postdiagnosis cancer pathway. The aim was to gain insight into the research landscape of AI, specifically the breadth and quality of studies evaluating AI algorithms to support the cancer pathway. In doing so, we can identify potential barriers to implementation and future research needs.

## Methods

The systematic review was designed using the Preferred Reporting Items for Systematic reviews and Meta-analyses. Studies published between 1 January 2013 and 1 May 2023 were searched on two online databases using PubMed (inclusive of Medline) and Embase. The following search terms were used to identify suitable publications: “Artificial Intelligence or Machine Learning or Deep Learning or Neural Network and Cancer or Tumour or Malignant”. The full search strategy used is available in the online supplemental data (online supplemental appendix A).

### Inclusion criteria

Studies in the postdiagnostic care pathway, evaluating validated AI algorithms to assess the efficacy or quality of the AI and/or its efficiency in patient workflows; adult solid organ malignancies or multiple tumour sites (this may include haematological malignancies), written in English and focused on human adults. Publications were accepted if they were prospective in nature, including (phase I–IV clinical trials), case-control studies and observational studies.

### Exclusion criteria

All publications that are retrospective or focused solely on haematological or paediatric malignancies are excluded. Review articles, letters, abstracts, conferences proceedings, editorials, preclinical studies, trial protocols and all studies published in non-English language are also excluded. Any studies in the development or validation phase and those training or evaluating an AI tool on retrospective datasets from the same institution were excluded.

### Data selection

The titles and abstracts were assessed. All potential abstracts were identified for full-text review. The studies were initially selected by SM with AA to check and assess the excluded studies. SM and PYN extracted data from each study, assessing its quality and any uncertainty was reviewed by AA and RS.

### Data extraction

The data included in the extraction are as follows:

- Location of studies.
- Characteristics of study (funding, setting, research design and sample size).
- Tumour site.
- Purpose of AI and component of cancer care pathway where AI was applied.
- Outcome of evaluation.
- Time, cost and resource use analysis.

### Data analysis

PYN conducted a risk of bias quality assessment for all randomised controlled trials and non-randomised interventional studies, using the Risk of Bias Assessment of Randomised Controlled Trials (ROB-2) and Risk of Bias In Non-randomised Studies-of Interventions (ROBIN-I) quality assessment tools, respectively.

## Results

### Search strategy

12457 publications were initially identified in the PubMed (inclusive of Medline) and Embase database. 32 studies were selected after the abstracts and titles were screened. Of these, 17 were excluded because of (1) being in the development and validation phase, (2) not directly investigating an AI tool, (3) preclinical phase studies or (4) clinical trial protocols. 15 publications fulfilled the search criteria and are summarised in online supplemental table S1 (online supplemental appendix B).

**Figure 1:**
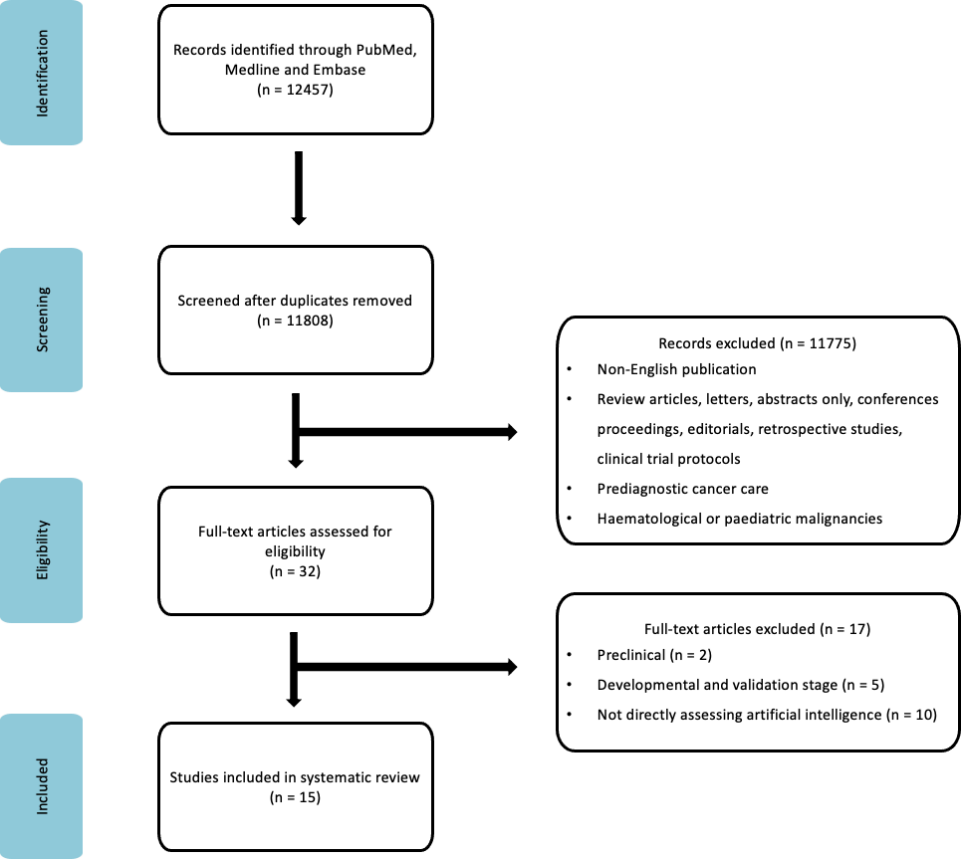
The Preferred Reporting Items for Systematic reviews and Meta-analyses flow chart of identification for articles for inclusion.

The search strategy is illustrated in figure 1.

### Tumour type, location, type of studies

The majority of studies focused on two or more tumour sites (n=8),^18^,^25^ with a particular emphasis on breast (n=8)^18^,^2023 24 26 27^ and gastrointestinal cancer (n=8).^18^,^2428^ Other cancer types included prostate (n=5),^20 22 25 29 30^ gynaecological (n=4)^20 21 23 24^ as well as thyroid and head and neck (n=5).^18 19 24 25 31^

Most studies were published in the USA (n=9).^18^,^2426 32^ Other published countries included Canada (n=3),^25 29 30^ China (n=2)^27 28^ and South Korea (n=1).^31^

All the included studies were prospective and described AI in the postdiagnostic cancer care pathway. Almost half were randomised control trials (n=7),^18^,^2126 29 31^ while the other half were observational studies (n=8).^22^,^2527 28 30 32^

### Types of AI investigated and clinical pathways

#### Clinician behaviour

Six studies focused on the use of AI to influence the patients’ or clinicians’ behaviour.^19 20 23 26 27 31^ Two studies identified whether using a machine learning algorithm predicting 180-day mortality and identifying high-risk patients with different cancer types, influences a clinicians’ decision to instigate serious illness conversations (SIC) and advanced care planning. In both studies, there was a significant increase in SIC from 3.4% to 13.5%.^19 23^ In addition, there was a decrease in the rates of systemic treatments used in patients approaching the end of life from 10.4% to 7.5%.^19 23^ Both studies were limited due to single healthcare electronic record. A further study assessed whether the artificial Clinical Decision Support System (CDSS) can change clinical treatment decisions in patients with breast cancer.^27^ The CDSS is an AI-based software that can make treatment recommendations to oncologists, based on clinical data drawn from clinical records.^27^ This study showed that treatment decisions changes in 5% of patients and more likely in hormone-positive or stage 4 disease (first-line treatment).^27^

#### Patient behaviour

AI can impact a patients’ behaviour, particularly with lifestyle measures such as exercise.^20 26^ A pilot study conducted over a 4-week period illustrated that an AI-based voice-coaching programme increased the average step-count by 3568.9 steps/day for overweight, physically inactive cancer survivors, compared with the control group 2160.6 steps/day (p<0.05).^20^ This study was limited by the small number of participants (n=42) and short follow-up period of 4 weeks.^20^ A second study assessed whether machine learning can provide accurate estimates of physical activity (PA), as there tends to be discrepancies between self-reporting and accelerometer data.^26^ This study showed that self-report and machine learning provided similar PA estimates at baseline (mean difference=11.5 min/day) and the mean difference of PA change for the cut-point versus machine learning methods was 5.1 min/day for intervention group and 2.9 in controls.^26^ Lastly, AI can be used to improve a patients’ understanding of their disease and improve the informed consent process for thyroid surgery.^31^ A deep neural network was used to design a personalised three-dimensional thyroid model.^31^ The group in the experimental arm showed a better understanding of their disease process, as well as the benefits and risks of thyroid surgery.^31^ However, the study was not blinded, which may have introduced an element of bias.^31^

#### Survival

Two studies assessed whether a machine learning can accurately predict prognosis in patients with advanced cancer.^22 23^ The Number of active tumors (“N”), Eastern Cooperative Oncology Group performance status (“E”), albumin (“A”) and primary tumor site (“T”) (NEAT) model provided better accurate prognostic predictions which was statistically significant, compared with experienced oncology physicians and nurses.^22^ However, its generalisability is limited due to it being single-site study.^22^ On the contrary, the other study, Manz *et al* demonstrated the feasibility and clinical validation of a machine learning for real-time short-term prognosis of patients with cancer across 18 centres under a single academic health system.^23^

#### Treatment

Four studies assessed AI in radiotherapy and brachytherapy.^25 29 30 32^ A machine learning brachytherapy treatment planning system for prostate cancer was tested in a prospective clinical trial and showed non-inferiority, in comparison with manual planning for the dose measured at implantation and 30 days.^29^ In addition, there was a significant reduction in treatment planning time.^29^ Similarly, a study focused on an AI auto-segmentation radiotherapy planning system for patients with non-small cell lung cancer with nodal involvement, showed a 65% reduction in segmentation time (p<0.0001).^32^ However, the major limitations were the lack of PET scan imaging for the radiotherapy planning, which is a common clinical application available for radiotherapy planning in lung cancer cases.^32^ A study conducted in China developed and tested an AI RAdioPathomics Integrated preDiction System.^28^ This tool predicted complete response in patient with locally advanced rectal cancer undergoing neoadjuvant chemoradiotherapy, based on pretreatment radiopathomic images with high accuracy.^28^ However, no demographic details were input into the model, which may improve its performance. A Canadian study trialled a fully workflow-integrated, machine learning-based radiotherapy planning software for patients with prostate cancer. Its outcome highlighted that AI acceptability by clinicians in real life differed from its retrospective evaluation^30^ (21% decrease in selection of peer-reviewed quantitatively superior machine learning radiotherapy plans by the clinicians at the simulation vs deployment phase, 92% vs 71%). Lastly, another Canadian study evaluated the performance of implemented deep learning-based auto-segmentation for central nervous system, head and neck and prostate cancer radiotherapy planning into the workflow. It concluded that the deep learning-based auto-segmented plans required minimal subjective (mean editing score ≤2) and objective edits (mean Dice similarity coefficient (DSC) and 95% Hausdorff distance (HD) was ≥0.90 and ≤2.0 mm) and resulted in a positive user experience.^25^

Within surgery, a trial assessed the use of a machine learning model to predict surgical case duration in gynae-oncology and colorectal surgical cases.^21^ This machine learning algorithm was better in predicting surgical case times, compared with the surgeons (p<0.03).^21^ This may enable better allocation of clinical resources and reduce patient waiting times.^21^ However, its accuracy was dependent on the correct data input and the system could only provide short-term predictions, 24 hours prior to the planned surgery.^21^

Lastly, a machine learning algorithm was trialled in the USA to identify high-risk patients with different solid organ malignancies undergoing radiotherapy or chemoradiotherapy.^24^ This algorithm identified patients likely to require acute care during their treatments and suggested twice-weekly on-treatment reviews.^24^ Twice-weekly evaluation reduced rates of acute care from 22.3% to 12.3% (p=0.02).^24^ Its main limitation is that it was conducted in a single centre.

### Quality assessment of studies

#### Randomised controlled trials

All studies involving randomised controlled trials were assessed using the ROB-2 quality assessment tool.^33^ The outcomes of the quality assessment are summarised in table 1.

**Table 1:** Risk of Bias Assessment of Randomised Controlled Trials (ROB-2)

Study	Domain 1	Domain 2		Domain 3	Domain 4	Domain 5	Overall risk of bias
	Risk of bias arising from the randomisation process	Risk of bias due to deviations from the intended interventions (effect of assignment to intervention)	Risk of bias due to deviations from the intended interventions (effect of adhering to intervention)	Missing outcome data	Risk of bias in measurement of the outcome	Risk of bias in selection of the reported result	
Manz *et al*^18^	Low	Low (unblinded participants)	Low	Low	Low	Low	Low
Manz *et al*^19^	Low	Low (unblinded participants)	Low	High risk (missing data for secondary outcome)	Low	Low	High risk (unpredictable)
Hassoon *et al*^20^	Low	Low (unblinded participants)	Low	Low	Low	Low	Low
Strömblad *et al*^21^	Low	Some concerns (Hawthorne’s effect and unequal arm size after exclusion postrandomisation)	Low	Low	Low	Low	Some concerns (unpredictable)
Seok *et al*^31^	Low	Low (unblinded participants)	Low	Low	Low	Low	Low
Nicolae *et al*^29^	Low	Low	Low	Low	Low	Low	Low
Nelson *et al*^26^	Low	High risk (per-protocol analysis with 13% dropout rate)	Low	High risk (13% missing outcome data)	Low	Low	High risk (unpredictable)

The risk of bias for each domain of the study is colour-coded. High risk of bias: red; moderate risk of bias: orange and low risk of bias: green.

Four out of seven randomised controlled trials (57.1%) were rated as having a low overall risk of bias across all five domains of risk of bias using the ROB-2 tool.^18 20 29 31^ Some concerns of risk of bias from effects of assignment to intervention were noted for Strömblad *et al*,^21^ due to 9.5% exclusion (n=72/755) postrandomisation.^19 21^ Manz *et al* was judged to have an overall high risk of bias specifically for their secondary outcomes end-of-life care and hospice enrolment, as they were missing 16.2% (n=229/1417) and 39.6% (n=569/1417) of outcome data, respectively due to study team’s dependence on hospital cancer registry for data collection and patients’ eligibility to hospices.^19 26^ Nelson *et al* was rated as having a high risk of bias due to their per-protocol analysis with 13% missing data (n=44/333), which have an unpredictable effect on the outcome.^26^

### Observational studies

All studies involving non-randomised interventions were assessed using the ROBINS-I quality assessment tool.^34^ The outcomes of the quality assessment are summarised in table 2.

**Table 2:** The Risk of Bias In Non-randomised Studies-of Interventions (ROBIN-I) assessment

Study	Risk of bias due to confounding	Risk of bias in selection of participants into the study	Risk of bias in classification of interventions	Risk of bias due to deviations from the intended interventions	Risk of bias due to missing data	Risk of bias in measurement of the outcome	Risk of bias in selection of the reported result	Overall risk of bias
Kao *et al*^22^	Low	Low	Low	Low	Low	Low	Low	Low
Manz *et al*^23^	Low	Low	Low	Low	Serious (missing ECOG status, excluded from analysis)	Low	Moderate (multiple subgroup analysis)	Serious
Hong *et al*^24^	Serious (unadjusted for confounding variables)	Low	Low	Low	Low	Low	Low	Serious
Feng *et al*^28^	Low	Low	Low	Low	Low	Low	Low	Low
Xu *et al*^27^	Serious (small sample size unadjusted for user acceptability of AI and clinical experience)	Low	Low	Low	Low	Low	Moderate (multiple subgroup analysis)	Serious
Hosny *et al*^32^	Low	Low	Low	Low	Low	Low	Low	Low
McIntosh *et al*^30^	Low	Low	Low	Low	Low	Low	Low	Low
Wong *et al*^25^	Low	Low	Low	Low	Low	Serious (unblinded participants with two subjective outcome measures)	Low	Serious

The risk of bias for each domain of the study is colour-coded. High risk of bias: red; moderate risk of bias: orange and low risk of bias: green.

AI, artificial intelligence; ECOG, Eastern Cooperative Oncology Group.

Half of the non-randomised studies of intervention were rated as having low overall risk of bias, meaning their risk is comparable to that of a well-conducted randomised trial.^22 28 30 32^ The overall risk of bias of Manz *et al* was deemed serious due to missing data and subsequent exclusion of patients with missing data from analysis.^23 24^ Hong *et al* was judged to have serious risk of bias, as the baseline characteristics of their cohorts were not adjusted to confounders, such as comorbidities, cancer diagnosis, age, gender and other relevant prognostic factors, which could significantly impact their outcomes, specifically emergency care attendance and hospital admission during cancer treatment.^24 27^ Xu *et al* looking at the effect of machine learning powered Clinical Decision Support System, suffered from serious risk of bias, as it relied on a small sample size of oncologists, who were not assessed for user acceptability of AI and adjusted for clinical experience prior to intervention.^25 27^ Wong *et al* did not blind the reviewers of the contours as to the source of the segmentation (deep learning-based or manual segmentation). The results of the study therefore suffered from a serious risk of bias in favour of the intervention arm.^25^

## Discussion

**Figure 2:**
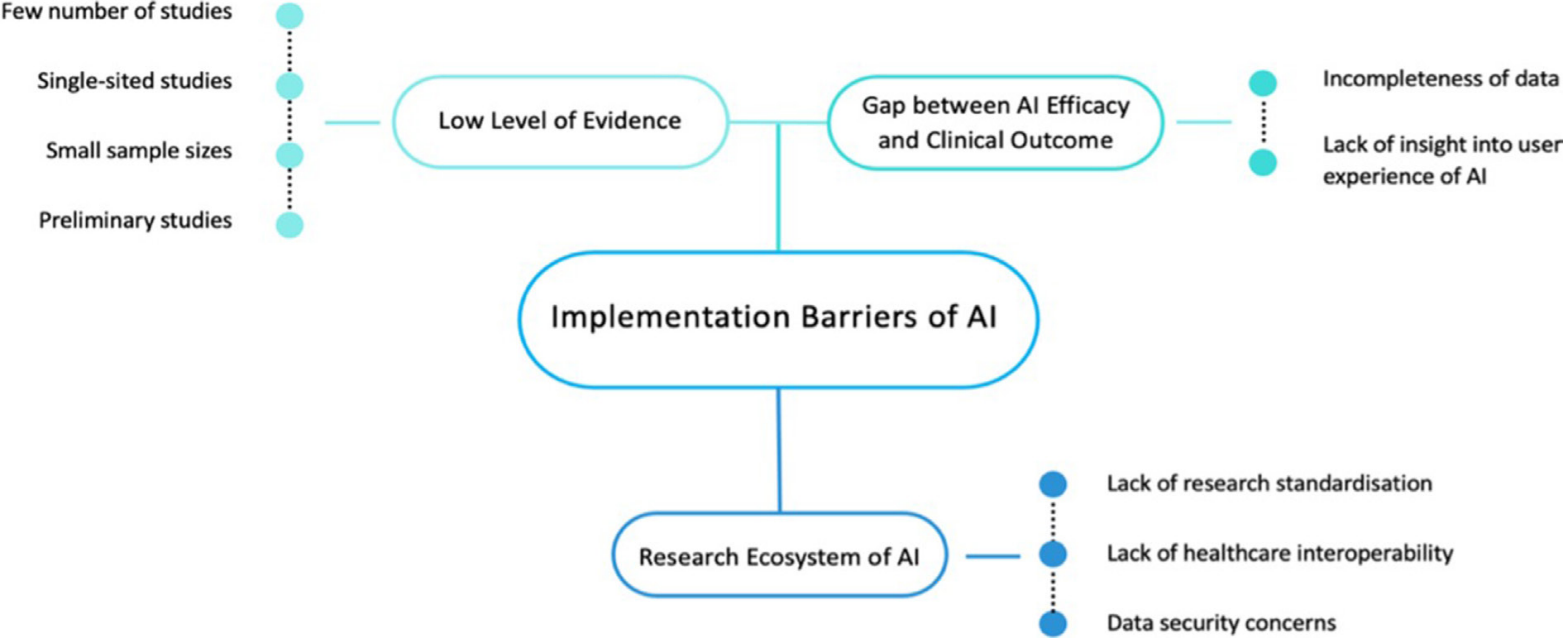
Implementation barriers of artificial intelligence (AI).

There is a concern that the clinical impact of AI may be limited or have piecemeal adoption because of the lack of robust evidence demonstrating its efficacy and cost-effectiveness. In this systematic review of all published studies evaluating the role of AI in the postcancer diagnostic pathway, we found only 15 studies that met our study criteria. The studies were predominantly single-centre studies with small sample sizes of ≤50 patients and no studies were conducted in the low-income and middle-income country setting. Overall, most oncology AI research remains at an experimental stage without prospective clinical validation or deployment due to implementation barriers illustrated in figure 2.^35 36^

### Low level of evidence

Most studies were in the pilot stage, that is, first time clinical deployment within tight trial parameters with relatively short follow-ups. Consequently, the clinical impact of AI in a real-world setting on integration into the healthcare system remains unknown. In addition, the design and conduct of the studies differed in quality, compromising the robustness of the evidence produced. Half of the studies were randomised controlled trials and the remaining half were observational. Quality assessment for risk of bias, using the ROB-2 and ROBIN-I tools for randomised controlled trials and observational studies, respectively, revealed that there were either at least some concerns or serious risk of bias in nearly half of the studies (n=6, 42.9%), due to unadjusted confounding variables, missing data, selective reporting and inappropriate method of analysis.

Most studies were conducted at a single clinical site (n=9, 64.3%), thus limiting its generalisability and external validity.^20,2325 26 28 30 31^ This potentially signalled technical difficulties in designing and developing studies across multiple sites due to a lack of interoperability among hospitals. Both adoption of unified data formats such as Fast Healthcare Interoperability Resources, and consistent clinical coding in electronic healthcare record, as promoted by the Minimal Common Oncology Data Elements in the USA, are necessary to overcome this hurdle to facilitate AI implementation, as demonstrated by multicentre, single health system studies conducted by Manz *et al*.^37 38^ The sample size of studies varied but given the context, some were too small (sample size ≤50) to provide sufficient power and precision for clinical deployment of the AI.^20 29 30 32^ Additional efforts to develop AI-ready data infrastructures rely on ontology approaches, such as the Operational Ontology for Oncology, to standardise real-world data for use in training or testing of novel algorithms.^39^

Two examples of large scale, high-quality prospective evaluation of AI tools in real-world setting were published by Dembrower *et al*^40^ and Lång *et al*^41^ to validate AI-supported mammogram screening for breast cancer in Sweden.^40 41^

### Gap between AI efficacy and clinical outcome

First, the measured metrics of the AI tool used in research might not directly translate into clinical benefits. The term ‘AI chasm’ was coined to reflect this phenomenon.^42^ To illustrate, serious illness conversations (SIC) were used as surrogates for goal-concordant care and less aggressive end-of-life care, assuming that SIC would improve concordance with advanced care plans and influence clinicians’ approach towards end-of-life care, such as avoiding prescription of systemic treatment close to death.^18^ However, when the same team used different end points with the same AI tool it found no effect on patients’ hospice enrolment, hospital length of stay, inpatient death or end-of-life intensive care unit admission. Thus, a robust clinical evaluation using indicators that are intuitive to clinicians and reflect the quality of care is essential. In the development and evaluation stage, the validity of the indicators should be based on its meaning and relevance to both clinicians and patients, that is, does the end point represent an important measure of quality or outcome relevant to patients.^43^ A difference in the indicator should also reflect a difference in the quality of care, with a specific direction reflecting better quality.^43^ Delphi techniques could be adopted to gain consensus among key stakeholders on the most important outcomes.^44^ An excellent example of AI studies with valid and technically specific indicators is the ongoing prospective ARtificial intelligenCe-based radiotHERapY (ARCHERY) study. This international prospective evaluation has been designed to independently evaluate according to a prespecified protocol the clinical acceptability (based on contouring and dosimetric parameters) of AI-based radiotherapy treatment planning for cervical, head and neck and prostate cancers. Alongside this, time and human resource savings have been estimated as well as a budget impact analysis.^45^

Second, most AI tools were calibrated using retrospective datasets. Low quality, incomplete and/or discrepant data in the clinical setting can negatively impact its real-world clinical performance.^10^,^12^ This is highlighted in the study by Strömblad *et al*,^21^ where their algorithm that predicts the duration of surgery was compromised in cases where the actual surgery undertaken in theatre deviated from the presurgical plan.^21^

Third, all existing studies, except for Hosny *et al*^32^ and Wong *et al*,^25^ did not explore the experience of AI users, be it healthcare providers or patients.^25 32^ This lack of insight may not affect the clinical outcome when the AI was only trialled by a small number of users over a short period of time, as demonstrated by Hassoon *et al* and Xu *et al*, but it poses uncertainties regarding the user acceptability, feasibility and sustainability of the AI tool in the long run and when it is scaled.^20 27^ The efficacy gap between retrospective simulation and real-life deployment of AI due to user acceptability was highlighted by McIntosh *et al*.^30^ They observed a 21% decrease in clinicians’ selection of AI-generated radiotherapy plans between simulation and treatment deployment even though the plans were deemed superior by blind expert reviews.^30^ To overcome this shortfall, implementation frameworks such as the Consolidated Framework for Implementation Research should be embedded in early stage research design to assess a range of contextual factors that hinder or facilitate the adoption of AI as a complex intervention, and to inform implementation strategies that may best address contextual determinants, such as clinicians’ bias.^46^

Fourth, the economics of AI implementation, in terms of time, cost and resources, were largely overlooked. Only two studies by Nicolae *et al*^29^ and Hosny *et al*^32^ went beyond in silico validation of AI to include a time-saving analysis, while one study by Hong *et al*^24^ analysed the potential resource saved using AI.^24 29 32^ Robust implementation validation should be encouraged in future studies as it is crucial to identify the downstream consequences of AI implementation in health systems, including the structure of care, process of care and workforce planning. This is particularly important in resource-limited countries, as their healthcare systems may not be mature enough to adopt AI tools. Equity consideration of AI interventions was also absent in all studies. To close the translational gap for real-life AI application in different resource settings, frameworks, such as Reach, Effectiveness, Adoption, Implementation and Maintenance, are recommended by WHO to help structure the implementation research.^17^

### Research ecosystem for AI

Our review showed that different methodologies were used to demonstrate efficacy in AI interventions of the same type, that serve the same purpose, indicating that there was a lack of standardisation in the evaluation and validation of AI. Although the area under the curve of the receiver operating characteristics is a popular statistical measure of the performance of machine learning models, it is often not sufficient to prove the clinical efficacy of AI.

Beyond statistical findings, the development environment (ie, clinical setting from which data used to train the model are generated), operational environment (ie, environment in which AI is deployed in including integration with health record system and infrastructure required) and human-AI interaction should be included in the research protocol to allow transparent and more holistic evaluation of an AI intervention, as recommended by the Standard Protocol Items: Recommendations for Interventional Trials - Artificial Intelligence (SPIRIT-AI) extension to the Consolidated Standards of Reporting Trials guidelines.^47^ Output data and reporting should also be tailored based on the type and purpose of AI. For example, an AI-driven image analytical tool should be reported using a class-activation map to visualise pixels that had the greatest influence on the predicted class.^46^ For predictive AI models, researchers should refer to the Transparent Reporting of a Multivariable Prediction Model for Individual Prognosis or Diagnosis-Machine Learning and Standards for Reporting Diagnostic Accuracy Studies-Artificial Intelligence, which were newly developed.^48 49^ Of the 15 studies included, only 2 studies used their relevant up-to-date guidelines, Transparent Reporting of a Multivariable Prediction Model for Individual Prognosis or Diagnosis.^23 28^

In terms of ethical and regulatory approval, data security is a concern as health data are often sensitive, private and stored in large volume. As an illustration, the AI voice coaching trialled in the study by Hassoon *et al*^20^ monitored the users’ day-to-day PA to provide feedback on their advice.^20^ Confidential data, as such, require safe methods of collection, storage and usage through a secure data server, and battles against data breach and sabotage will require consistent vigilance, investment and legislature protection.

### Recommendations

To overcome the triad of insufficient clinical evidence, efficacy-outcome gap and new research ecosystem of AI that currently hinders the implementation of AI in oncology, we propose the recommendations to key stakeholders as mentioned in table 3.

**Table 3:** Action plans tailored to stakeholders for addressing specific issues

Current issues	Action plans	Stakeholders
Lack of interoperability between hospitals	Engage with medical informatics system vendors to facilitate integration of AI and secure data storage	Healthcare providers
Lack of validation of AI quality/efficacy	Conduct tests using independent external data to validate, optimise and audit AI efficacy	
Lack of standardisation in evaluation and validation of AI	Develop and mandate the use of standard oncology terminologies and ontologies Set the standards required to evaluate the performance of AI-based tools systematically Establish an up-to-date regulatory and legal frameworks for different AI based on implementation risks	Commissioners and regulators
Lack of integration of implementation science framework	Establish consensus regarding trial protocol involving AI to standardise reporting Conduct AI studies that validate patient-centred outcomes and cost/time/resource effectiveness Promote implementation science research to learn optimal methods to AI deployment in cancer care	Academics and healthcare providers
Lack of workforce training	Level up on knowledge of AI and basics of medical informatics Prepare for disruption and adapt to changes in nature of work with the integration of AI	Healthcare professionals

AI, artificial intelligence.

More funding is required overall, but more importantly, a larger proportion of funding should be dedicated to implementation science research of AI. In the UK, the NHS AI Lab, led by the National Institute of Health Research and NHS England, creates a community space for clinicians, data scientists, healthcare providers and regulators. It also facilitates funding and implementation of AI in clinical environment through AI Health and Care Award for AI at different phases of trials.^50^ Such multidisclipinary collaboration backed by funding should be expanded to bridge the chasm between AI and clinical implementation in routine clinical cancer care.

### Strengths and limitations of review

The strengths of our review lie in our comprehensive yet targeted inclusion criteria (specifically prospective studies in postdiagnostic cancer care) and robust methodology triangulated by systematic review and quality assessment. We also provided stakeholder-specific action plans by learning from successful examples available in a wider context.

By design, our review excluded prospective AI studies in cancer diagnostics, which make up the majority of AI research and are the most advanced on the frontier, such as studies by Dembrower *et al*^40^ and Lång *et al*.^41^ As a result, we were unable to analyse these studies, some of which were deployed in real-world environments and across different healthcare ecosystems. Additionally, previous computational or predictive models that were not classified as AI/machine learning/deep learning/neural networks were excluded, but may have parallels or considerations that should be considered when addressing our recommendations.

## Conclusion

AI is a fast-growing technology with immense potential to reshape cancer care and pathways beyond cancer diagnostics. Despite the exponential growth in AI research into postdiagnostic cancer care, only a small fraction of AI tools have undergone prospective clinical evaluation and concerns were highlighted regarding the size of the study, the breadth of participants and the study conduct. Resource, cost-effectiveness and time-saving analyses of AI were largely missing; as were qualitative surveys on user acceptability, feasibility and sustainability of AI.

Future work should focus on multicollaborative AI implementation research co-developed by academics, healthcare providers, commissioners and patients, in accordance with up-to-date research guidelines and local health systems. They should focus on holistically evaluating AI technologies and determining their readiness for safe, feasible and efficient clinical deployment.

## Supplementary material

10.1136/bmjonc-2023-000255 online supplemental file 1

## Data Availability

All data relevant to the study are included in the article or uploaded as supplementary information.

## References

[1] Chua IS, Gaziel-Yablowitz M, Korach ZT (2021). Artificial intelligence in oncology: path to implementation. Cancer Med.

[2] Somashekhar SP, Sepúlveda M-J, Puglielli S (2018). Watson for oncology and breast cancer treatment recommendations: agreement with an expert Multidisciplinary tumor board. Ann Oncol.

[3] Mak RH, Endres MG, Paik JH (2019). Use of crowd innovation to develop an artificial intelligence–based solution for radiation therapy targeting. JAMA Oncol.

[4] Luchini C, Pea A, Scarpa A (2022). Artificial intelligence in oncology: Current applications and future perspectives. Br J Cancer.

[5] McKinney SM, Sieniek M, Godbole V (2020). International evaluation of an AI system for breast cancer screening. Nature.

[6] Eloy C, Marques A, Pinto J (2023). Artificial intelligence–assisted cancer diagnosis improves the efficiency of Pathologists in Prostatic biopsies. Virchows Arch.

[7] Senior K (2023). NHS embraces AI-assisted radiotherapy technology. Lancet Oncol.

[8] Cristiano S, Leal A, Phallen J (2019). Genome-wide cell-free DNA fragmentation in patients with cancer. Nature.

[9] Cabral BP, Braga LAM, Syed-Abdul S (2023). Future of artificial intelligence applications in cancer care: A global cross-sectional survey of researchers. Curr Oncol.

[10] Kelly CJ, Karthikesalingam A, Suleyman M (2019). Key challenges for delivering clinical impact with artificial intelligence. BMC Med.

[11] Crawford K, Calo R (2016). There is a blind spot in AI research. Nature.

[12] Chen I, Frederik J, Sontag D (2018). Why is my Classifier discriminatory?. Adv Neural Inf Process Syst.

[13] Topol EJ (2019). High-performance medicine: the convergence of human and artificial intelligence. Nat Med.

[14] Zhou N, Zhang C-T, Lv H-Y (2019). Concordance study between IBM Watson for oncology and clinical practice for patients with cancer in China. Oncologist.

[15] Hutson M (2018). Artificial intelligence faces reproducibility crisis. Science.

[16] Aristidou A, Jena R, Topol EJ (2022). Bridging the chasm between AI and clinical implementation. The Lancet.

[17] Jandoo T (2020). WHO guidance for Digital health: what it means for researchers. Digit Health.

[18] Manz CR, Parikh RB, Small DS (2020). Effect of integrating machine learning mortality estimates with behavioral Nudges to Clinicians on serious illness conversations among patients with cancer: A stepped-wedge cluster randomized clinical trial. JAMA Oncol.

[19] Manz CR, Zhang Y, Chen K (2023). Long-term effect of machine learning–triggered behavioral Nudges on serious illness conversations and end-of-life outcomes among patients with cancer: A randomized clinical trial. JAMA Oncol.

[20] Hassoon A, Baig Y, Naiman DQ (2021). Randomized trial of two artificial intelligence coaching interventions to increase physical activity in cancer survivors. NPJ Digit Med.

[21] Strömblad CT, Baxter-King RG, Meisami A (2021). Effect of a predictive model on planned surgical duration accuracy, patient wait time, and use of Presurgical resources: A randomized clinical trial. JAMA Surg.

[22] Kao J, Zucker A, Urso M (2022). Improving survival prognostication in patients with metastatic cancer through clinical judgment. Anticancer Res.

[23] Manz CR, Chen J, Liu M (2020). Validation of a machine learning algorithm to predict 180-day mortality for outpatients with cancer. JAMA Oncol.

[24] Hong JC, Eclov NCW, Dalal NH (2020). System for high-intensity evaluation during radiation therapy (SHIELD-RT): A prospective randomized study of machine learning–directed clinical evaluations during radiation and Chemoradiation. J Clin Oncol.

[25] Wong J, Huang V, Wells D (2021). Implementation of deep learning-based auto Segmentation for radiotherapy planning structures: a Workflow study at two cancer centers. Radiat Oncol.

[26] Nelson SH, Natarajan L, Patterson RE (2019). Physical activity change in an RCT: comparison of measurement methods. Am J Health Behav.

[27] Xu F, Sepúlveda M-J, Jiang Z (2020). Effect of an artificial intelligence clinical decision support system on treatment decisions for complex breast cancer. JCO Clin Cancer Inform.

[28] Feng L, Liu Z, Li C (2022). Development and validation of a Radiopathomics model to predict pathological complete response to Neoadjuvant Chemoradiotherapy in locally advanced Rectal cancer: a Multicentre observational study. Lancet Digit Health.

[29] Nicolae A, Semple M, Lu L (2020). Conventional vs machine learning–based treatment planning in prostate Brachytherapy: results of a phase I randomized controlled trial. Brachytherapy.

[30] McIntosh C, Conroy L, Tjong MC (2021). Clinical integration of machine learning for curative-intent radiation treatment of patients with prostate cancer. Nat Med.

[31] Seok J, Yoon S, Ryu CH (2021). A personalized 3d-printed model for obtaining informed consent process for thyroid surgery: A randomized clinical study using a deep learning approach with mesh-type 3d modeling. J Pers Med.

[32] Hosny A, Bitterman DS, Guthier CV (2022). Clinical validation of deep learning Algorithms for radiotherapy targeting of non-small-cell lung cancer: an observational study. Lancet Digit Health.

[33] Sterne JAC, Savović J, Page MJ (2019). Rob 2: a revised tool for assessing risk of bias in randomised trials. BMJ.

[34] Sterne JA, Hernán MA, Reeves BC (2016). ROBINS-I: a tool for assessing risk of bias in non-randomised studies of interventions. BMJ.

[35] Hamamoto R, Suvarna K, Yamada M (2020). Application of artificial intelligence technology in oncology: towards the establishment of precision medicine. Cancers (Basel).

[36] Kann BH, Hosny A, Aerts H (2021). Artificial intelligence for clinical oncology. Cancer Cell.

[37] Mandel JC, Kreda DA, Mandl KD (2016). SMART on FHIR: a standards-based, Interoperable Apps platform for electronic health records. J Am Med Inform Assoc.

[38] mCODE:Minimal Common Oncology Data Elements.

[39] Mayo CS, Feng MU, Brock KK (2023). Operational Ontology for oncology (O3): A professional society-based, Multistakeholder, consensus-driven Informatics standard supporting clinical and research use of real-world data from patients treated for cancer. *International Journal of Radiation Oncology*Biology*Physics*.

[40] Dembrower K, Crippa A, Colón E (2023). Artificial intelligence for breast cancer detection in screening Mammography in Sweden: a prospective, population-based, paired-reader, non-inferiority study. Lancet Digit Health.

[41] Lång K, Josefsson V, Larsson A-M (2023). Artificial intelligence-supported screen reading versus standard double reading in the Mammography screening with artificial intelligence trial (MASAI): a clinical safety analysis of a randomised, controlled, non-inferiority, single-blinded, screening accuracy study. Lancet Oncol.

[42] Keane PA, Topol EJ (2018). With an eye to AI and autonomous diagnosis. NPJ Digit Med.

[43] Geary R, Knight H, Carroll F (2018). A Step‐Wise approach to developing indicators to compare the performance of maternity units using hospital administrative data. *BJOG*.

[44] Avery KNL, Chalmers KA, Brookes ST (2018). Development of a core outcome set for clinical effectiveness trials in Esophageal cancer resection surgery. Ann Surg.

[45] Aggarwal A, Court LE, Hoskin P (2023). ARCHERY: a prospective observational study of artificial intelligence-based radiotherapy treatment planning for Cervical, head and neck and prostate cancer – study protocol. BMJ Open.

[46] Damschroder LJ, Reardon CM, Widerquist MAO (2022). The updated Consolidated framework for implementation research based on user feedback. Implement Sci.

[47] Cruz Rivera S, Liu X, Chan A-W (2020). The SPIRIT-AI and CONSORT-AI working group, et al. guidelines for clinical trial protocols for interventions involving artificial intelligence: the SPIRIT-AI extension. Nat Med.

[48] Collins GS, Reitsma JB, Altman DG (2015). Transparent reporting of a multivariable prediction model for individual prognosis or diagnosis (TRIPOD): the TRIPOD statement. Circulation.

[49] Sounderajah V, Ashrafian H, Aggarwal R (2020). Developing specific reporting guidelines for diagnostic accuracy studies assessing AI interventions: the STARD-AI steering group. Nat Med.

[50] The artificial intelligence in health and care award. https://transform.england.nhs.uk/ai-lab/ai-lab-programmes/ai-health-and-care-award/.

