# Correction: Prospective evaluation of artificial intelligence (AI) applications for use in cancer pathways following diagnosis: a systematic review

**DOI:** 10.1136/bmjonc-2023-000255corr1

**Published:** 2025-05-15

 Macheka S, Ng PY, Ginsburg O, *et al.* Prospective evaluation of artificial intelligence (AI) applications for use in cancer pathways following diagnosis: a systematic review. *BMJ Oncol* 2024;3:e000255.

Article type has been changed from ‘Review’ to ‘Original Research’.

