# Supplementary material for: Prospective evaluation of artificial intelligence (AI) applications for use in cancer pathways following diagnosis: a systematic review
Source: BMJ Oncol. 2024 May 10;3(1):e000255. doi: 10.1136/bmjonc-2023-000255 (PMC11235004; doi:10.1136/bmjonc-2023-000255)
Supplement: online supplemental file 1 [file bmjonc-2023-000255supp001.pdf]

Supplementary Data

1. Appendix A- Full Search Strategy

Search: (((machine learning[Title/Abstract]) OR (artificial intelligence [Title/Abstract])) OR (deep learning[Title/Abstract])) OR (neural network[Title/Abstract]))) AND (cancer[Title/Abstract]) OR (malignant[Title/Abstract]) OR (tumour[Title/Abstract]) Filters: Clinical Study, Clinical Trial, Clinical Trial Protocol, Clinical Trial, Phase I, Clinical Trial, Phase II, Clinical Trial, Phase III, Clinical Trial, Phase IV, Comparative Study, Controlled Clinical Trial, Observational Study, Pragmatic Clinical Trial, Randomized Controlled Trial, 10 years, Humans, English.

2. Appendix B- Summary Table of Included Studies

Table S1: Summary Table of Included Studies

Authors, Year	Tumour Site(s)	Study Type	Number of Patients and Location	Type Of Artificial Intelligence and Care Pathway	Study Aims	Study Outcomes and Funding source	Analysis of Artificial Intelligence (Cost, Time, Resource)
Manz et al, 2020(18)	Multiple: breast, central nervous system, gastrointestinal, genitourinary, lymphoma, melanoma, myeloma, and thoracic or head and neck	RCT<br><br>Prospective<br><br>Initial 16-week results	14607 patients<br><br>USA - multi-centre, single health system	Machine Learning algorithm predicting 180-day mortality<br><br>Behaviour- serious illness conversations	Does Machine Learning algorithm predicting 180-day mortality alter clinical behaviour on Serious illness conversations (SIC) in patient encounters?	SIC conducted among 1.3% control group vs 4.6% in intervention group. Machine learning predicted mortality can positively influence the rate of SIC among patients and oncology clinicians<br><br>Funding: Penn Center for Precision Medicine Accelerator Fund and award T32 from the National Institute of General Medical Sciences, and the University of Pennsylvania Health System	No formal analysis.
Manz et al, 2023(19)	Multiple: breast, central nervous system, gastrointestinal, genitourinary, lymphoma, melanoma, myeloma, and thoracic or head and neck	RCT<br><br>Prospective<br><br>40-week results	20506 patients<br><br>USA – multi-centre, single health system	Machine Learning algorithm predicting 180-day mortality<br><br>Behaviour- serious illness conversations	Does Machine Learning algorithm predicting 180-day mortality alter clinical behaviour on Serious illness conversations (SIC) and end of life outcomes among patients with cancer?	The machine learning algorithm increased SIC conducted high risk patients from 3.4% to 13.5% and decreased rates of end-of-life systemic therapy from 10.4% to 7.5%<br><br>Funding: NIH grant 5K08CA263541 and the Penn Center for Precision Medicine (Drs Manz and Parikh) and NIH grant K08CA263541 (Dr Parikh).	No formal analysis.
Hassoon et al, 2021(20)	Multiple: Breast, Prostate, Lung, Colorectal, Cervical and Oral	RCT<br><br>Pilot study<br><br>Prospective	42 patients<br><br>USA - Single Centre	AI coaching software using digital voice assist or data smart text messaging<br><br>Behaviour - Impact on physical activity	To assess whether AI voice coaching vs text messages and control (written literature) has an impact of step count	Physical activity increased in the AI based arm with a net difference = 3568.9 steps/day (95% CI: 1483–5655), P value <0.001] compared to control arm, and [net difference = 2160.6 steps/day (95% CI: 11–4310), P value 0.049] compared to SmartText. AI-based coaching may be a practical to increase physical activity in sedentary cancer survivors	No formal analysis.<br><br>Acknowledged challenge with computational resource required to scale AI.

						Funding: Institution from the American Heart Association (20SFRN35380046 and COVID19-811000), PCORI (ME-2019C1-15328), National Institutes of Health (P01 HL108800), Aetna Foundation, the David and June Trone Family Foundation, the Pollin Digital Innovation Fund, PJ Schafer Cardiovascular Research Fund, CASCADE FH, and Google.	
Strömblad et al, 2021(21)	Multiple: Gynaecological and Colorectal	RCT<br><br>Prospective	683 patients<br><br>USA - Single Centre	Machine Learning model predicting surgical case duration<br><br>Treatment - Surgery	Assess whether machine learning model can accurately predict surgical case duration	Machine learning prediction model identified reduction in error for scheduling surgery duration estimates (MAE, $p=0.03$), improving patient waiting time and resources.<br><br>Funding: National Institutes of Health/National Cancer Institute (Cancer Center support grant P30 CA008748).	No formal analysis.<br><br>Resource intensive as some each sample requires 300 data points, of which some require manual entry. Acknowledged need for implementation workflow to electronic health record.
Seok et al, 2021(22)	Thyroid	RCT –<br><br>Prospective	60 patients<br><br>South Korea - Single Centre	Neural network 3D thyroid model<br><br>Behaviour – Understanding of disease, indications and risk of surgery	Compare the degree of a patient's understanding of their disease and surgery with or without the use of a personalized 3D thyroid model	The group provided with personalized 3D-printed models showed significant improvement in all four categories (general knowledge, benefits and risks of surgery, and satisfaction; all $p < 0.05$). This may be utilised for improving a patient's understanding during consent.<br><br>Funding: National Cancer Center in Republic of Korea [grant numbers NCC2031580-1 and NCC2010241-2].	No formal analysis.<br><br>The mean cost and production time of the 3D printed models were included and acknowledged as limitations.<br>.
Kao et al, 2022(23)	Multiple: Lung, Prostate, Colorectal, Breast	Cohort<br><br>Prospective	73 patients<br><br>USA - Single Centre	AI prognostic Model<br><br>Survival	To assess whether experienced clinicians can accurately predict survival in advanced cancer patients compared to AI prognostic model	The accuracy of nursing predictions was 61.6% and the accuracy of physician predictions was 60.3% (*p*=0.85). Experienced clinicians can modestly supplement the accuracy of a validated model to predict survival in patients with advanced cancer.<br><br>Funding: Not stated	No formal analysis.

Manz et al, 2020(24)	Multiple: Breast, GI, Thoracis, GU, Gynaecologic, Leukaemia, Lymphoma, Melanoma, Myeloma, Neuro-oncology	Cohort<br><br>Prospective	24 582 patients<br><br>Single health system- 18 centres<br>USA – Multicentre (national)	Machine Learning algorithm to predict 180 day mortality<br><br>Survival	To validate a Machine Learning algorithm embedded in electronic health care records to generate 180 day mortality risk	The AUC was 0.89 (95% CI, 0.88-0.90), the AUPRC was 0.34, and the scaled Brier score was 0.29. ML algorithm to predict short-term mortality has good prospective validity and compares favourably with existing prognostic indices.<br><br>Funding: Penn Center for Precision Medicine Accelerator Fund and grant T32 GM075766-14 from the National Institute of General Medical Sciences	No formal analysis.
Nicolae et al, 2020(25)	Prostate	Prospective<br><br>Non-Inferiority, phase 1 RCT	41 Patients<br><br>Canada – Single Centre	Machine Learning based prostate implant planning algorithm<br><br>Treatment - Brachytherapy	Evaluate non inferiority of Day 30 dosimetry between machine learning planning and conventional manual planning for prostate brachytherapy	No significant difference in preimplant or day 30 dose in prostate or rectum between machine learning and conventional techniques. There was a significant difference in planning time. This has potential to offer time saving and resource allocation.<br><br>Funding: TELUS Ride for Dad granting agency	Time analysis included- conventional planning time of 43.13 ± 58.70 min (6.00–315 min) vs machine-learning-based planning time of 2.38 ± 0.96 min (1.15–4.44 min), p <0.05.<br><br>Acknowledged benefit in reducing staff required for planning.
Hong et al, 2020(26)	Multiple: GI, Breast, Genitourinary, Gynaecologic, Head and Neck, Thoracic, Bone	Prospective<br><br>Quality Improvement<br><br>Single Centre	963 patients<br><br>USA - Single Centre	Machine Learning algorithm to identify high risk patients<br><br>Treatment	To assess whether machine learning can identity high risk patients and direct twice weekly clinical evaluation to reduce acute care during visits	Twice weekly evaluation reduced rates of acute care from 22.3% to 12.3% P=0.02. Machine learning can triage patients undergoing RT and CRT, to influence clinical management and reduce health care costs.<br><br>Funding: Not stated	Resource analysis included- twice weekly evaluation reduced rates of acute care from 22.3% to 12.3% P=0.02<br><br>Cost analysis is underway.
Feng at al, 2022(27)	Rectal cancer	Observational<br><br>Prospective<br><br>.	115 patients<br><br>China - Multicentre (national)	Artificial intelligence RAdioPathomics Integrated preDiction System (RAPIDS)<br><br>Treatment Response	Develop and validate an artificial intelligence RAdioPathomics Integrated preDiction System (RAPIDS) for the prediction of pathological complete response to neoadjuvant chemo-radiotherapy in	RAPIDS had an AUC of 0·812 (95% CI 0·717–0·907), sensitivity of 0·888 (0·728–0·999), specificity of 0·740 (0·593–0·886), NPV of 0·929 (0·862–0·995), and PPV of 0·512 (0·313–0·710).<br><br>Funding: National Natural Science Foundation of China; Youth Innovation Promotion Association of the Chinese Academy of Sciences.	No formal analysis.

					patients with locally advanced rectal cancer using pretreatment MRI and haematoxylin and eosin (H&E)-stained biopsy slides.		
Nelson et al, 2019(28)	Breast cancer	RCT Prospective	333 participants<br><br>USA - Single Centre	To assess whether machine learning algorithm estimate physical activity<br><br>Behaviour	To quantify the agreement between self-report, standard cut-point accelerometer, and machine learning accelerometer estimates of physical activity (PA), and examine how agreement changes over time among older adults in an intervention setting.	At baseline, self-report and machine learning provided similar PA estimates (mean difference = 11.5 min/day) unlike self-report and standard cut-points (mean difference = 36.3 min/ day) Specifically, the mean difference of PA change for the cut-point versus machine learning methods was 5.1 min/day for intervention group and 2.9 in controls, whereas it was ≥ 24.7 min/day for other comparisons.<br><br>Funding: National Cancer Institute	No formal analysis.
Xu et al, 2020(29)	Breast cancer	Cross Sectional<br><br>Prospective	1977 patients<br><br>China - Multicentre (national)	Artificial Intelligence based CDSS (Clinical Decision Support System)<br><br>Behaviour	To examine the impact of a clinical decision support system (CDSS) on breast cancer treatment decisions and adherence to National Comprehensive Cancer Center (NCCN) guidelines.	Treatment decisions changed in 105 (5%) of 1,977 patients and were concentrated in those with hormone receptor (HR)–positive disease or stage IV disease in the first-line therapy setting (73% and 58%, respectively). Logistic regressions showed that decision changes were more likely in those with HR-positive cancer (odds ratio [OR], 1.58; P , .05) and less likely in those with stage IIA (OR, 0.29; P , .05) or IIIA cancer (OR, 0.08; P , .01).<br><br>Funding: IBM Watson Health	No formal analysis.

Hosny et al, 2022(30)	Non Small Cell Lung Cancer	Observational Study<br><br>Retrospective and Prospective	28 patients (end user testing)<br><br>USA – Single Centre	Deep Learning methods for segmenting NSCLC and Lymph nodes on CT images<br><br>Treatment: Radiotherapy	To develop a multifaceted strategy for the clinical validation of deep learning models for radio- therapy targeting in non-small cell lung cancer	Models showed an improvement over the interobserver benchmark (multi-delineation dataset; VD 0·91 [IQR 0·83–0·92], p=0·0062; SD 0·86 [0·71–0·91], p=0·0005), and were within the intra-observer benchmark. AI assistance led to a 65% reduction in segmentation time (5·4 min; p<0·0001) and a 32% reduction in interobserver variability (SD; p=0·013).<br><br>Funding: US National Institutes of Health and EU European Research Council	Time analysis- 65% reduction in segmentation time (5·4 min; p<0·0001).
McIntosh et al, 2021(31)	Prostate Cancer	Observational<br><br>Retrospective and Prospective	50 patients (retrospective)<br><br>50 patients (prospective)<br><br>Canada- Single centre	Machine learning algorithm for therapeutic curative-intent radiation therapy treatment planning<br><br>Treatment: Radiotherapy	To evaluate random forest algorithm for therapeutic curative-intent radiation therapy (RT) treatment planning for prostate cancer with full integration into the clinical workflow	89% of ML-generated RT plans were considered clinically acceptable and 72% were selected over human-generated RT plans in head-to-head comparisons.<br><br>While ML RT plan acceptability remained stable between the simulation and deployment phases (92 versus 86%), the number of ML RT plans selected for treatment was significantly reduced (83 versus 61%, respectively, p=0.02). These findings highlight that retrospective or simulated evaluation of ML methods, even under expert blinded review, may not be representative of algorithm acceptance in a real-world clinical setting when patient care is at stake.	Time analysis- RT planning using ML reduced the median time required for the entire RT planning process by 60.1% (118 to 47 h).

Wong et al, 2021 (32)	Central nervous system (CNS), head and neck (H&N) and prostate cancer	Observational<br><br>Prospective	551 patients<br><br>Canada- two centres	Implementation of deep-learning auto segmentation for CNS, H&N and prostate cancer radiotherapy planning into the workflow<br><br>Treatment: Radiotherapy	To evaluate the performance of implemented deep-learning based auto segmented contour (DC) models in the clinical radiotherapy (RT) planning workflow subjectively and objectively as well as report on user experience	The majority of Organs At Risk (OAR) DCs required minimal edits subjectively (mean editing score $\leq$ 2) and objectively (mean DSC and 95% HD was $\geq$ 0.90 and $\leq$ 2.0 mm). Mean OAR satisfaction score was 4.1 for CNS, 4.4 for H&N, and 4.6 for prostate structures.	No formal time analysis – subjective reporting of time savings with using DCs, before and after auto-segmentation implementation.
